# From assembly inference to co-occurrence organization: conservation contexts are associated with gut bacterial variation in the endangered *Gymnocypris przewalskii*

**DOI:** 10.3389/fmicb.2026.1875592

**Published:** 2026-07-09

**Authors:** Jiangqi Qu, Yanfei Wu, Hongfang Qi, Li Chen, Ying Luo, Yang Wang, Qingjing Zhang

**Affiliations:** 1 Beijing Key Laboratory of Fishery Biotechnology, Fisheries Science Institute, Beijing Academy of Agriculture and Forestry Sciences, Beijing, China; 2 National Engineering Research Center for Freshwaters, Fisheries Science Institute, Beijing Academy of Agriculture and Forestry Sciences, Beijing, China; 3 Provincial Key Laboratory of Breeding and Protection of Qinghai Lake Naked Carp of Qinghai, Qinghai Lake Naked Carp Rescue Center, Xining, Qinghai, China; 4 Hebei Ocean and Fisheries Science Research Institute, Qinhuangdao, Hebei, China

**Keywords:** conservation contexts, endangered *Gymnocypris przewalskii*, environmental filtering, gut bacteria, stochastic and deterministic processes

## Abstract

**Introduction:**

Conservation programs increasingly rely on in-situ and ex-situ interventions to prevent the loss of endangered species; however, it remains unclear whether such interventions preserve host-associated microbial organization. Understanding how conservation contexts influence gut microbiome structure and function is therefore essential for evaluating their ecological effectiveness.

**Methods:**

We analyzed gut bacterial communities of the endangered high-altitude fish *Gymnocypris przewalskii* across wild, in-situ conservation, and *ex-situ* conservation contexts using 16S rRNA gene sequencing. Analyses included host morphometric covariate assessment, alpha- and beta-diversity statistics, co-occurrence network analysis, PICRUSt2-based functional prediction, and null-model-based community assembly inference.

**Results:**

Gut bacterial communities were consistently dominated by Proteobacteria, with Firmicutes, Bacteroidetes, Actinobacteria, and Chloroflexi as secondary taxa. Alpha diversity showed no significant differences among groups, whereas beta diversity revealed modest but significant compositional shifts associated with conservation context, independent of multivariate dispersion and host morphometric traits. Functional predictions indicated shifts in pathways related to carbohydrate, energy, lipid, amino acid, terpenoid, polyketide metabolism, secondary metabolite biosynthesis, and xenobiotic degradation. Co-occurrence networks exhibited context-dependent restructuring: wild communities showed highest connectivity and positive interactions, *in-situ* networks were most modular and sparsest, and *ex-situ* communities showed the highest proportion of negative interactions. Null-model analyses indicated dominance of stochastic processes across all groups, with highest drift and stochasticity in *in-situ* populations, while *ex-situ* communities showed stronger deterministic influence and homogeneous selection.

**Discussion:**

These results demonstrate that conservation contexts are associated not only with taxonomic turnover but also with functional potential shifts, altered co-occurrence topology, and changes in community assembly processes. Within the constraints of an observational and site-confounded design, our findings suggest that microbiome-informed conservation assessment should move beyond diversity metrics and incorporate compositional, functional, network, and assembly-based perspectives when evaluating conservation outcomes in endangered aquatic hosts.

## 1 Introduction

Global biodiversity is undergoing rapid and unprecedented decline under the combined pressures of habitat fragmentation, climate change, and intensified anthropogenic disturbances (Tonkin et al., 2026). These stressors not only reduce species abundance and genetic diversity, but also alter ecological interactions and host-associated bacterial communities that may contribute to host nutrition, immune regulation, and environmental responsiveness (Segan et al., 2016; Sandor et al., 2022). In response, conservation strategies have increasingly incorporated a spectrum of interventions, ranging from *in-situ* protection to *ex-situ* management and assisted translocation, aiming to preserve biodiversity and restore ecosystem functionality (Volis, 2017; Rodríguez-Rodríguez and Martínez-Vega, 2022). However, the biological consequences of these conservation settings are still commonly evaluated using demographic, genetic, or habitat-based indicators, whereas host-associated bacterial responses remain less well-integrated into conservation assessment. This gap is particularly relevant for endangered aquatic species, whose gut bacterial communities may respond rapidly to changes in water chemistry, diet, habitat complexity, and microbial exposure.

Host-associated microbiota, particularly gut bacterial communities, are now recognized as integral components of animal physiology and ecological adaptation (Bornbusch and Muletz-Wolz, 2026). These communities contribute to nutrient metabolism, immune regulation, and environmental stress tolerance, forming a dynamic interface between the host and its environment (Diwan et al., 2023). Because aquatic animals are continuously exposed to surrounding water, sediment, and diet-derived microbial inputs, their gut bacterial communities can respond sensitively to changes in water chemistry, salinity, temperature, diet composition, stocking density, and habitat structure (Gao et al., 2023). Habitat transitions, including captivity, translocation, and artificial propagation, may therefore be associated with shifts in gut bacterial composition and predicted functional potential (Korpita et al., 2023; Singh et al., 2024; Kanika et al., 2025). Such bacterial patterns may provide complementary ecological information for evaluating conservation outcomes, especially for endangered aquatic species for which long-term physiological monitoring and destructive sampling are constrained.

Bacterial community assembly is governed by underlying ecological processes and interaction dynamics rather than solely by compositional variation (Bittleston, 2024). Community assembly theory provides a useful framework for evaluating the relative contributions of stochastic processes (e.g., ecological drift and dispersal) and deterministic processes (e.g., environmental filtering and selection) in shaping microbiota structure (Stegen et al., 2015; Zhou and Ning, 2017). In parallel, co-occurrence network analysis has emerged as an important approach for describing bacterial association patterns and community organization, including connectivity, modularity, and the balance between positive and negative associations (Barberán et al., 2012; Faust and Raes, 2012; Berry and Widder, 2014). Environmental disturbance and habitat simplification can alter microbial association networks and assembly patterns, potentially affecting community organization and functional redundancy (Coyte et al., 2015; Banerjee et al., 2018). However, despite these theoretical and methodological advances, the integration of community assembly, co-occurrence organization, and predicted functional potential remains limited in conservation studies of endangered aquatic organisms.

The Qinghai-Tibet Plateau, often referred to as the “Third Pole,” represents a unique and fragile ecosystem characterized by extreme environmental conditions and high levels of endemism. Qinghai Lake, the largest inland saltwater lake in China, serves as a critical ecological refuge and biodiversity hotspot within this region (Lu et al., 2022; Li et al., 2024). The endemic fish *Gymnocypris przewalskii* (Tibetan naked carp) has evolved remarkable physiological adaptations to high-altitude, saline–alkaline environments (Tian et al., 2019; Linlin et al., 2024). Nevertheless, due to its slow growth, low fecundity, and narrow ecological niche, this species remains highly vulnerable to environmental perturbations and anthropogenic pressures (Walker et al., 1996; Xiong et al., 2010). Conservation efforts, including fishing bans, artificial propagation, and habitat restoration, have contributed to population recovery (Chen et al., 2012; Weng et al., 2023; Dudgeon, 2024). However, how different conservation contexts are associated with gut bacterial communities and their ecological organization in this endangered fish remains poorly understood.

Given their high sensitivity to environmental variation and their close association with host physiology, gut bacterial communities have emerged as promising bioindicators for assessing ecosystem health and conservation outcomes (Degregori et al., 2021; Bertucci et al., 2022; Huang et al., 2024). Unlike traditional monitoring approaches that focus primarily on population size or habitat characteristics, gut-bacteria-based indicators can capture rapid, integrative responses to environmental change at the physiological and ecological levels (Trevelline et al., 2019). However, most existing studies have focused on taxonomic composition or diversity patterns (Gan et al., 2026), with less attention to inferred assembly processes, co-occurrence organization, and predicted functional potential across different conservation contexts. This gap limits our ability to interpret gut bacterial variation beyond compositional change and to develop microbiome-informed, but cautiously framed, tools for conservation assessment and management.

In this study, we used the endangered high-altitude fish *Gymnocypris przewalskii* as a model to examine gut bacterial communities across three representative conservation contexts: wild population, *in-situ* conservation, and *ex-situ* conservation. We first characterized bacterial alpha diversity, beta diversity, and dominant taxonomic composition, and then assessed whether community differences remained associated with conservation context after accounting for measured host morphometric traits. We further evaluated PICRUSt2-predicted functional pathway profiles, explored bacterial co-occurrence network organization and robustness, and inferred assembly categories using βNTI and RC_bray_ null models. Through this stepwise framework, we asked whether gut bacterial communities differed among conservation contexts in terms of diversity, composition, predicted function, co-occurrence organization, and inferred assembly, and whether these bacterial features could provide complementary information for conservation assessment of *G. przewalskii*. This study provides a gut-bacteria-based perspective for evaluating conservation-associated bacterial variation in an endangered aquatic species.

## 2 Materials and methods

### 2.1 Sampling design and ethical statement

This study was conducted from July to August 2022 across three representative conservation contexts for the endangered Tibetan naked carp *G. przewalskii*: wild population, *in-situ* conservation, and *ex-situ* conservation. A total of 27 sexually mature male individuals were sampled, with nine individuals per conservation context. Only males were destructively sampled to reduce variation associated with sex and reproductive status and to avoid potential impacts on reproductive females of this endangered species. Because *G. przewalskii* is a nationally protected endangered species, sample size was constrained by conservation regulations, ethical permissions, and the need to minimize disturbance to protected populations.

For each individual, total length (L, cm) and body weight (W, g) were measured, and Fulton's condition factor was calculated as K = 100 × W/L^3^ (Froese, 2006). All sampled fish were sexually mature adults. Exact individual age records were unavailable for wild fish; therefore, wild individuals were described as sexually mature adults, with approximate adult status inferred from body size, sexual maturity, and field observations. *In-situ* and *ex-situ* individuals were 3-year-old sexually mature adults according to breeding and management records. Group-level morphometric and developmental-stage data are provided in Supplementary Table S1.

Within each conservation context, the nine fish were randomly selected and treated as individual-level biological replicates. However, independent site-level replicates were not available. The wild group was sampled from one approved monitoring area at the Buha River inflow of Qinghai Lake, the *in-situ* group from the Qinghai Lake Naked Carp Rescue and Conservation Center within the native distribution region, and the *ex-situ* group from one breeding facility in Hebei Province. Therefore, conservation context was confounded with site-level environmental factors, including geography, altitude, salinity, water chemistry, temperature regime, and diet. Accordingly, the study was interpreted as an observational comparison of conservation contexts rather than a replicated experimental test of conservation strategy effects.

All experimental procedures complied with institutional animal care and use guidelines and the Regulations of the People's Republic of China on the Protection of Aquatic Wildlife. Prior to sampling, fish were anesthetized with MS-222 (tricaine methanesulfonate; 150 mg L^−1^). Under sterile conditions, intestinal contents from the mid-to-distal intestine were aseptically collected using sterile instruments to minimize external contamination. All samples were immediately flash-frozen in liquid nitrogen after collection and subsequently stored at −80 °C until DNA extraction.

### 2.2 Definition of conservation contexts and environmental setting

To provide environmental context for interpreting gut bacterial variation, the three sampling groups were defined as representative conservation contexts differing in environmental naturalness, spatial relocation, and management intensity: wild population, *in-situ* conservation, and *ex-situ* conservation. The wild group represented the natural high-altitude saline–alkaline lake-river ecosystem; the *in-situ* group represented a standardized artificial propagation and rearing program located within the native distribution region; and the *ex-situ* group represented a spatially relocated, lowland, freshwater breeding system under artificial management. These contexts differed in geography, altitude, water chemistry, salinity, temperature regime, and diet; therefore, environmental descriptions were used to contextualize gut bacterial community differences among conservation contexts. Detailed water physicochemical parameters and diet/feed information are provided in Supplementary Tables S2, S3.

#### 2.2.1 Wild group

Wild individuals were collected from the Buha River inflow region of Qinghai Lake, China (39.19 °N, 100.22 °E; altitude 3,200–3,260 m above sea level). Qinghai Lake is a high-altitude saline–alkaline lake ecosystem characterized by low temperature, high alkalinity, and relatively high salinity. During the sampling period, surface-water physicochemical parameters were measured *in situ* using a YSI EXO2 multiparameter instrument, and detailed values are provided in Supplementary Table S2. Fish in this habitat relied primarily on natural food resources, including phytoplankton, zooplankton, benthic invertebrates, algae, and organic detritus. Therefore, the wild group was used as the natural reference context for comparing gut bacterial communities across conservation settings.

#### 2.2.2 In-situ conservation group

The *in-situ* conservation group consisted of individuals bred and maintained at the Qinghai Lake Naked Carp Rescue and Conservation Center, located within the native distribution region of *G. przewalskii*. This context followed a standardized artificial propagation and rearing program within the species' native region. The conservation process included: (i) collection of wild broodstock during the spawning season; (ii) induced spawning and egg incubation in flow-through hatchery facilities; and (iii) rearing larvae and juveniles in recirculating ponds under standardized management conditions. Water physicochemical parameters were monitored during the sampling period and are summarized in Supplementary Table S2. Fish in this group were fed a specialized formulated feed for naked carp, with feed composition and nutritional information provided in Supplementary Table S3. The *in-situ* group was therefore used to represent a managed conservation context within the native distribution region.

#### 2.2.3 *Ex-situ* conservation group

The *ex-situ* conservation group consisted of individuals cultured at a breeding facility in Hebei Province, China (39.35 °N, 115.50 °E). Fish were reared in freshwater ponds under controlled aquaculture conditions with artificial aeration and scheduled feeding. Compared with the wild and *in-situ* contexts, the *ex-situ* context involved spatial relocation outside the native high-altitude region, reduced salinity, simplified habitat structure, and intensive artificial management. Water physicochemical parameters and feed composition are provided in Supplementary Tables S2, S3. Individuals were fed commercial formulated feed rather than natural food resources. Although *G. przewalskii* can tolerate a broad range of salinity conditions, the *ex-situ* system differed substantially from the native lake-river environment in geography, altitude, water chemistry, salinity, and diet. Therefore, this group was interpreted as a representative *ex-situ* conservation context rather than as an isolated effect of artificial management alone.

### 2.3 DNA extraction and 16S rRNA gene sequencing

Genomic DNA was extracted from intestinal contents of *Gymnocypris przewalskii* using the QIAamp Fast DNA Stool Mini Kit (Qiagen, Hilden, Germany) with minor modifications to improve DNA yield from high-altitude fish intestinal samples (Han et al., 2018). DNA concentration and purity were assessed using a NanoDrop 2000 spectrophotometer (Thermo Scientific, Wilmington, DE, USA), and all DNA samples were diluted to 1 ng·μL^−1^ prior to PCR amplification.

The V3–V4 hypervariable regions of the bacterial 16S rRNA gene were amplified using universal primers 338F and 806R (Zhou et al., 2024). PCR amplification was performed in triplicate for each sample to minimize amplification bias. Amplified products were verified by electrophoresis on 2% agarose gels and subsequently purified using the AxyPrep DNA Gel Extraction Kit (Axygen Biosciences, Union City, CA, USA). Purified amplicons were quantified using a QuantiFluor™-ST fluorometer (Promega, Madison, WI, USA), pooled in equimolar concentrations, and subjected to paired-end sequencing (2 × 300 bp) on the Illumina MiSeq platform (Illumina, San Diego, CA, USA). Raw sequencing data generated in this study were deposited in the NCBI Sequence Read Archive (SRA) under accession number SRP311066.

### 2.4 Sequence processing and taxonomic annotation

Raw sequencing reads were first demultiplexed according to barcode sequences and quality-filtered using Trimmomatic v0.39 (Bolger et al., 2014). Paired-end reads were subsequently merged using FLASH v1.2.11 (Magoč and Salzberg, 2011). Chimeric sequences were identified and removed using the UCHIME algorithm to obtain high-quality clean reads. Operational taxonomic units (OTUs) were clustered at 97% sequence similarity using UPARSE v11 (Edgar, 2013). Representative sequences from each OTU were taxonomically assigned against the SILVA 138 ribosomal RNA database using the RDP Classifier with a confidence threshold of 70%. To ensure comparability among samples, sequence datasets were rarefied to the minimum sequencing depth prior to downstream ecological analyses.

Although amplicon sequence variant approaches such as DADA2 and Deblur provide higher sequence resolution (Callahan et al., 2016), a 97% OTU-based workflow was retained for consistency and comparability with previous fish gut bacterial studies using similar OTU-based approaches, including studies on *Gymnocypris przewalskii* and other fish species (Wang et al., 2021; Bi et al., 2023). Because this study focused on community-level ecological analyses, including gut bacterial diversity, taxonomic composition, beta-diversity, co-occurrence organization, null-model-inferred assembly patterns, and predicted functional potential, rather than strain- or species-level sequence variants, the OTU-based workflow was used for community-level analyses. Given the lower taxonomic resolution of 97% OTU clustering compared with ASV-based methods, ecological interpretation was restricted mainly to OTU-, phylum-, and genus-level bacterial patterns, and species-level assignments were not used for ecological inference.

### 2.5 Microbial diversity analysis

Alpha diversity of gut bacterial communities was evaluated using the ACE, Chao1, and Shannon indices to characterize within-sample bacterial richness and diversity. Differences in alpha diversity among conservation contexts were evaluated using Kruskal-Wallis tests, given the limited sample size per group and potential deviations from normality. Beta diversity was evaluated using Bray-Curtis dissimilarity and weighted UniFrac distances (Lozupone and Knight, 2005). Community dissimilarities were visualized using non-metric multidimensional scaling (NMDS) and principal coordinates analysis (PCoA). Statistical significance of group separation was assessed using analysis of similarities (ANOSIM; Somerfield et al., 2021) and permutational multivariate analysis of variance (PERMANOVA), implemented in the vegan package in R. The PERMANOVA effect size was reported as *R*^2^ to indicate the proportion of total community variation explained by conservation context. To test whether significant PERMANOVA results could be influenced by unequal within-group dispersion, homogeneity of multivariate dispersion was assessed using PERMDISP based on distances to group centroids (Anderson, 2006). In addition, to evaluate whether measured host morphometric traits confounded bacterial community differences, total length, body weight, and Fulton's condition factor were included as covariates in Bray-Curtis PERMANOVA models. The covariate model was specified as: Bray-Curtis distance matrix ~ total length + body weight + condition factor + conservation context. This model was used to test whether conservation context remained associated with gut bacterial community composition after accounting for measured host morphometric traits.

Taxonomic composition was summarized and visualized at both the phylum and genus levels using the *ggplot2* package. Ternary plots were further applied to compare genus-level compositional differences among conservation habitats. To identify microbial taxa with significantly different relative abundances among groups, linear discriminant analysis effect size (LEfSe) analysis was performed (Segata et al., 2011). Taxa with an LDA score > 3.5 and adjusted *P* < 0.05 were considered significantly enriched and were described as genus-level indicators rather than definitive biomarkers.

### 2.6 PICRUSt2-based functional prediction and NSTI evaluation

Potential functional profiles of gut bacterial communities were predicted using PICRUSt2 (Douglas et al., 2020). This approach infers gene family abundances by placing bacterial taxa into a reference phylogenetic framework and linking them to closely related reference genomes. Predicted functional profiles were annotated against the Kyoto Encyclopedia of Genes and Genomes (KEGG) database, and metabolic pathways at KEGG Levels 2 and 3 were assigned to each sample to characterize microbial functional potential. Because PICRUSt2 predicts functional potential from 16S rRNA gene data rather than directly measuring gene expression, enzyme activity, metabolite production, or metabolic activity, all functional results were interpreted as predicted functional potential. The nearest sequenced taxon index (NSTI) was used to evaluate the average phylogenetic distance between query taxa and reference genomes (Langille et al., 2013; Douglas et al., 2020).

Sankey diagrams generated using the *ggalluvial* package in R were used to visualize the relative contributions of major functional categories and to illustrate pathway shifts associated with different conservation environments. To compare predicted functional profiles among conservation contexts, KEGG pathway abundances were normalized and stratified at Level 3. Statistical differences among groups were evaluated using Welch's *t*-test, and *p*-values were adjusted using the Benjamini-Hochberg false discovery rate correction. Pathways with adjusted *p*-values <0.05 were considered significantly enriched. To further explore associations between microbial taxonomy and predicted metabolic functions, Spearman's rank correlation analyses were conducted between dominant bacterial genera and significantly enriched KEGG pathways. Correlation matrices were visualized as clustered heatmaps using the *pheatmap* package in R. Strong correlations were defined as *|*ρ*|* > 0.6 and *P* < 0.01 and interpreted as associations between bacterial genera and predicted pathway profiles rather than direct functional interactions.

### 2.7 Co-occurrence network analysis and robustness assessment

Genus-level bacterial co-occurrence networks were constructed separately for each conservation context using Spearman's rank correlations. Before network construction, rare genera detected in fewer than 20% of samples or with mean relative abundance below 0.01% were removed to reduce sparsity (Berry and Widder, 2014). Pairwise Spearman's rank correlations were calculated among the retained genera within each conservation context, and edges were retained when the absolute Spearman correlation coefficient was |ρ| > 0.6 and *P* < 0.01 (Faust and Raes, 2012). Separate networks were generated using the *igraph* package in R and visualized in Gephi version 0.9.2. Nodes represented bacterial genera, and edges represented significant positive or negative co-occurrence associations. Network topology was summarized using node number, edge number, node degree, average path length, clustering coefficient, modularity, graph density, and positive-to-negative edge ratios to describe genus-level co-occurrence organization among conservation contexts (Barberán et al., 2012).

To evaluate whether observed network structures differed from random expectation, permutation-based randomization tests were performed following null-model-based network comparison approaches (Deng et al., 2012; Röttjers et al., 2021). For each conservation context, networks were reconstructed 1,000 times after randomizing sample labels or abundance profiles under the same filtering and correlation thresholds, and observed edge numbers were compared with randomized edge distributions (Röttjers et al., 2021). To assess edge-level robustness, bootstrap edge-recovery analysis was performed by resampling samples within each conservation context with replacement 1,000 times and reconstructing networks under the same thresholds. Edge recovery was calculated as the number of observed edges recovered in the bootstrap network divided by the total number of observed edges. Mean edge-recovery ratios and 95% confidence intervals were reported (Deng et al., 2012). To partially account for compositional constraints in relative-abundance data, centered log-ratio (CLR) transformed networks were also constructed as sensitivity analyses following compositional data analysis principles (Gloor et al., 2017).

### 2.8 Community assembly analysis

Null-model-based community assembly analysis was performed using the beta-nearest taxon index (βNTI) and the Raup-Crick metric based on Bray-Curtis dissimilarity (RC_bray_), following established microbial community assembly frameworks (Stegen et al., 2015; Zhou and Ning, 2017). βNTI values were calculated using the iCAMP package in R (Ning et al., 2019, 2020). Pairwise comparisons with βNTI > 2 were assigned to heterogeneous selection, whereas comparisons with βNTI <−2 were assigned to homogeneous selection. Pairwise comparisons with |βNTI| <2 were further partitioned using RC_bray_. When |βNTI| <2, comparisons with RC_bray_ > 0.95 were assigned to dispersal limitation, those with RC_bray_ <−0.95 were assigned to homogenizing dispersal, and those with −0.95 ≤ RC_bray_ ≤ 0.95 were assigned to drift or undominated stochastic processes. The relative contributions of homogeneous selection, heterogeneous selection, dispersal limitation, homogenizing dispersal, and drift/undominated processes were summarized for each conservation context and visualized using ggplot2.

### 2.9 Statistical analysis

All statistical analyses were conducted using R software version 4.3.0. Normality was assessed using the Shapiro–Wilk test, and homogeneity of variance was assessed using Levene's test. For host morphometric traits, including total length, body weight, and Fulton's condition factor, one-way analysis of variance (ANOVA) followed by Tukey's *post hoc* test was used when assumptions of normality and homogeneity of variance were met. Kruskal–Wallis tests were used for non-normally distributed variables or as non-parametric sensitivity analyses.

Alpha-diversity indices were compared among conservation contexts using Kruskal–Wallis tests. Beta-diversity differences were evaluated using ANOSIM and PERMANOVA, and homogeneity of multivariate dispersion was tested using PERMDISP. To evaluate potential confounding effects of host morphometric traits, Bray-Curtis PERMANOVA models including total length, body weight, and condition factor as covariates were used. For PICRUSt2-predicted functional profiles, differences in KEGG pathway abundances among groups were evaluated using Welch ANOVA followed by pairwise Welch tests with BH correction, and *P*-values were adjusted using the Benjamini–Hochberg false discovery rate correction. Network robustness was assessed using permutation-based randomization tests and bootstrap edge-recovery analyses. Statistical significance was defined as *P* < 0.05 unless otherwise specified. Graphical visualizations were generated using R packages including *ggplot2, vegan, igraph, ggalluvial*, and *pheatmap*.

## 3 Results

### 3.1 Gut bacterial diversity, beta-diversity patterns, and host morphometric covariates

High-throughput 16S rRNA gene sequencing generated a total of 1,338,262 high-quality reads across all samples after quality filtering. To ensure comparability, all samples were rarefied to 29,314 sequences per sample, resulting in the identification of 5,164 OTUs at 97% sequence similarity. Sequencing coverage exceeded 99.8% for all samples, indicating sufficient depth to comprehensively capture gut bacterial diversity.

**Figure 1:**
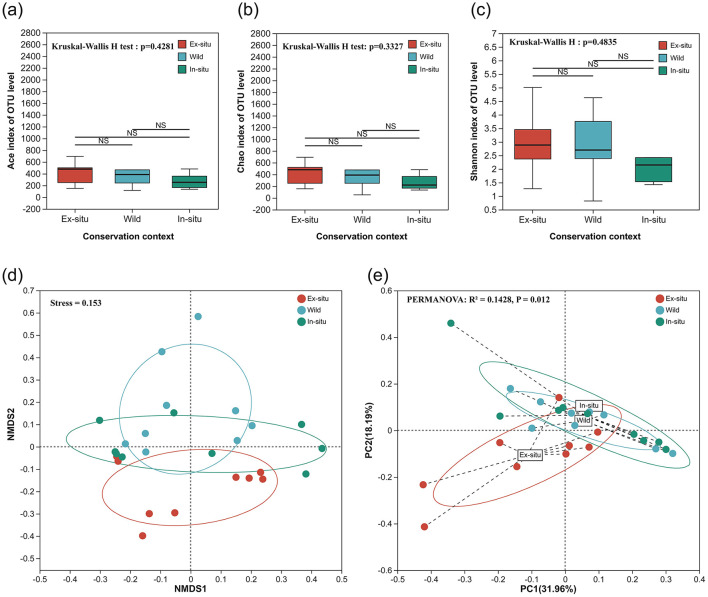
Gut bacterial diversity and community structure across conservation contexts. **(a–c)** Alpha diversity indices, including ACE, Chao1, and Shannon, showed no significant differences among the wild, *in-situ*, and *ex-situ* groups. **(d)** NMDS based on Bray–Curtis dissimilarity showed partial group separation with overlapping distributions. **(e)** PCoA based on weighted UniFrac distances also showed partially overlapping group distributions.

Alpha diversity, assessed using ACE, Chao1, and Shannon indices, did not differ significantly among the wild, *in-situ*, and *ex-situ* groups (ACE: *P* = 0.4281; Chao1: *P* = 0.3327; Shannon: *P* = 0.4835; Figures 1a–c; Supplementary Table S4), indicating comparable within-sample bacterial richness and diversity across conservation contexts. Beta-diversity analyses indicated modest but significant differences in gut bacterial community composition among conservation contexts. NMDS based on Bray–Curtis dissimilarity showed partial group separation with overlapping distributions and a stress value of 0.153 (Figure 1d). PCoA based on weighted UniFrac distances showed a similar pattern of partially overlapping group distributions (Figure 1e). ANOSIM indicated weak-to-moderate group separation (*R* = 0.1762, *P* = 0.01), and PERMANOVA showed that conservation context explained 14.28% of total bacterial community variation (*R*^2^ = 0.1428, *P* = 0.012; Supplementary Table S5). PERMDISP was not significant (*F* = 0.127, *P* = 0.898; Supplementary Table S5), indicating that the PERMANOVA result was not primarily driven by unequal within-group dispersion.

Host morphometric and developmental-stage data are summarized in Supplementary Table S1. Total length, body weight, and Fulton's condition factor differed significantly among conservation contexts (Supplementary Table S6). One-way ANOVA showed significant group differences in total length (*F* = 20.270, *P* = 6.99 × 10^−6^), body weight (*F* = 42.76, *P* = 1.23 × 10^−8^), and condition factor (*F* = 42.30, *P* = 1.36 × 10^−8^). Tukey *post hoc* comparisons showed that wild fish were significantly smaller and had lower condition factor than both *in-situ* and *ex-situ* fish, whereas *in-situ* and *ex-situ* fish did not differ significantly in these morphometric traits. To evaluate whether these host traits explained the observed bacterial community differences, Bray–Curtis PERMANOVA models were fitted with total length, body weight, and condition factor as covariates. The combined morphometric covariates were not significant (*F* = 1.256, R^2^ = 0.1408, *P* = 0.1624), whereas conservation context remained significant after accounting for these traits (*F* = 2.120, R^2^ = 0.1443, *P* = 0.0063; Supplementary Table S6C). Thus, the observed beta-diversity pattern was not fully explained by measured host morphometric differences.

### 3.2 Taxonomic composition and PICRUSt2-predicted functional profiles

**Figure 2:**
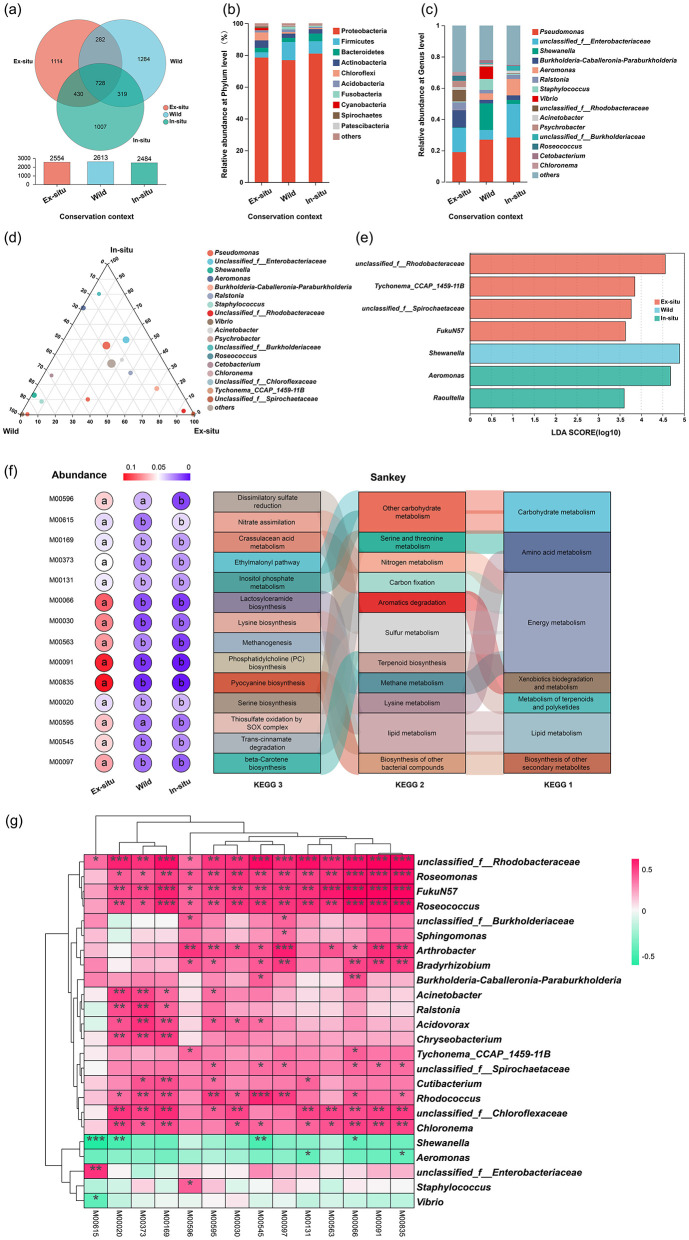
Taxonomic composition and PICRUSt2-predicted functional profiles of gut bacterial communities across conservation contexts. **(a)** Venn diagram showing shared and unique OTUs among wild, *in-situ*, and *ex-situ* groups. **(b)** Relative abundance of dominant bacterial phyla across groups. **(c)** Relative abundance of dominant bacterial genera in each group. **(d)** Ternary plot showing genus-level distribution patterns among conservation contexts. **(e)** LEfSe analysis identifying significantly enriched genera among groups. Taxa with LDA scores > 3.5 and adjusted *P* < 0.05 were considered significantly enriched. **(f)** PICRUSt2-predicted KEGG pathways across groups. Predicted pathways were classified according to KEGG categories. **(g)** Spearman correlation heatmap between dominant bacterial genera and significantly enriched predicted KEGG pathways/modules. Only significant correlations (|ρ| > 0.6, *P* < 0.01) are displayed.

Taxonomic annotation identified 5,164 OTUs assigned to 48 phyla, 122 classes, 329 orders, 591 families, and 1,285 genera across all samples. Venn diagram analysis showed that 728 OTUs were shared among the wild, *in-situ*, and *ex-situ* groups, whereas 1,284 OTUs were unique to the wild group, 1,007 OTUs were unique to the *in-situ* group, and 1,114 OTUs were unique to the *ex-situ* group (Figure 2a). Because the study used a 97% OTU-based workflow, taxonomic interpretation was focused on OTU-, phylum-, and genus-level patterns.

At the phylum level, Proteobacteria dominated gut bacterial communities across all conservation contexts, accounting for 76.81% in the wild group, 80.97% in the *in-situ* group, and 78.49% in the *ex-situ* group (Figure 2b; Supplementary Table S7). Firmicutes was the second most abundant phylum in the wild and *in-situ* groups, accounting for 11.41% and 7.75%, respectively, whereas Chloroflexi and Actinobacteria were relatively more abundant in the *ex-situ* group, accounting for 5.01% and 4.65%, respectively. Bacteroidetes accounted for 2.67%, 4.84%, and 2.85% in the wild, *in-situ*, and *ex-situ* groups, respectively.

At the genus level, *Pseudomonas* was the most abundant genus in both the wild and *in-situ* groups, accounting for 27.00% and 28.35%, respectively, and accounted for 18.95% in the *ex-situ* group (Figure 2c; Supplementary Table S7). *Unclassified_f_Enterobacteriaceae* was most abundant in the *in-situ* group, accounting for 21.44%, followed by 15.52% in the *ex-situ* group and 6.16% in the wild group. *Shewanella* was mainly represented in the wild group, accounting for 17.01%, compared with 2.57% in the *in-situ* group and 0.30% in the *ex-situ* group. *Burkholderia-Caballeronia-Paraburkholderia* accounted for 11.07% in the *ex-situ* group, compared with 2.94% in the *in-situ* group and 2.27% in the wild group. *Aeromonas* accounted for 10.46% in the *in-situ* group, compared with 4.17% in the wild group and 0.12% in the *ex-situ* group.Ternary plot analysis further illustrated genus-level distribution patterns among conservation contexts (Figure 2d).In the wild group, *Vibrio, Pseudomonas, Staphylococcus*, and *Shewanella* were positioned toward the wild vertex. In the *ex-situ* group, *Unclassified_f_Rhodobacteraceae, Unclassified_f_Spirochaetaceae, Tychonema_CCAP_1459-11B*, and *Unclassified_f_Chloroflexaceae* were mainly distributed toward the *ex-situ* vertex. In the *in-situ* group, Aeromonas and *Unclassified_f_Burkholderiaceae* were positioned toward the *in-situ* vertex. LEfSe analysis identified seven genera with significantly different relative abundances among conservation contexts (LDA score > 3.5, adjusted *P* < 0.05; Figure 2e; Supplementary Table S7). These enriched genera included *Unclassified_f_Rhodobacteraceae, Tychonema_CCAP_1459-11B, Unclassified_f_Spirochaetaceae, FukuN57, Shewanella, Aeromonas*, and *Raoultella*.

PICRUSt2-based prediction identified 14 KEGG pathways/modules that differed among groups (adjusted *P* < 0.05; Figure 2f; Supplementary Table S8). These predicted pathways were mainly assigned to carbohydrate metabolism, energy metabolism, lipid metabolism, amino acid metabolism, terpenoid and polyketide metabolism, secondary metabolite biosynthesis, and xenobiotic biodegradation and metabolism. Weighted NSTI values ranged from 0.0203 to 0.2069, with an overall mean of 0.0782 ± 0.0507. Group-level weighted NSTI values were 0.0787 ± 0.0290 for wild fish, 0.0710 ± 0.0690 for *in-situ* fish, and 0.0848 ± 0.0516 for *ex-situ* fish (Supplementary Table S8). Spearman correlation analysis was used to evaluate associations between dominant bacterial genera and significantly enriched predicted KEGG pathways/modules (Figure 2g). Several genera, including *Vibrio, Aeromonas*, and *Shewanella*, showed predominantly negative correlations with multiple predicted pathways, whereas *Unclassified_f_Rhodobacteraceae, Roseomonas, FukuN57, Roseococcus*, and *Unclassified_f_Chloroflexaceae* showed positive correlations with more than 70% of the significantly enriched predicted pathways/modules (|ρ| > 0.6, *P* < 0.01).

### 3.3 Co-occurrence network topology and robustness

**Figure 3:**
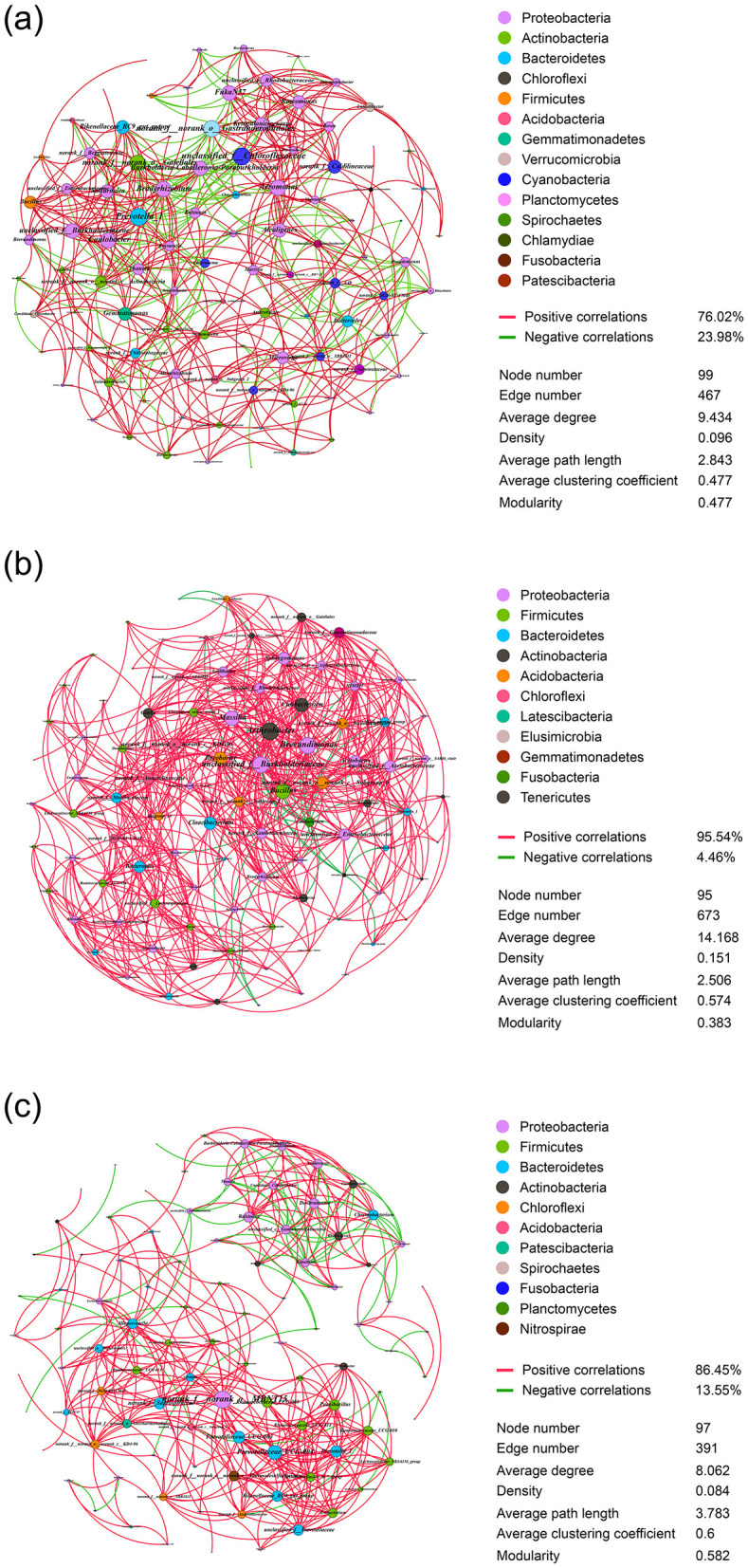
Genus-level bacterial co-occurrence networks across conservation contexts. Co-occurrence networks were constructed separately for the **(a)** *ex-situ*, **(b)** wild, and **(c)** *in-situ* groups using retained bacterial genera after rare-taxon filtering. Edges were retained when |ρ| > 0.6 and *P* < 0.01. Nodes represent bacterial genera, and node colors indicate bacterial phyla. Red and green edges indicate positive and negative co-occurrence associations, respectively. Node size is proportional to node degree. Network statistics shown in each panel include positive and negative association percentages, node number, edge number, average degree, graph density, average path length, average clustering coefficient, and modularity.

Genus-level bacterial co-occurrence networks were constructed separately for each conservation context using retained genera after rare-taxon filtering, with edges retained at |ρ| > 0.6 and *P* < 0.01. The three observed networks contained similar numbers of nodes, ranging from 95 to 99, but differed in edge number, connectivity, graph density, path length, modularity, and the balance between positive and negative associations (Figure 3; Supplementary Table S9). These metrics showed context-specific differences in genus-level co-occurrence organization, without supporting a simple monotonic gradient from wild to *in-situ* to *ex-situ*.

The wild network contained 95 nodes and 673 edges, including 643 positive and 30 negative associations. It had the highest edge number, average degree (14.168), graph density (0.151), and average clustering coefficient (0.574), together with the shortest average path length (2.506). Positive associations accounted for 95.54% of all edges, whereas negative associations accounted for 4.46%. These values showed that the wild network was the most connected of the three observed networks, with the highest proportion of positive co-occurrence associations and the shortest average path length. The *in-situ* network contained 97 nodes and 391 edges, including 338 positive and 53 negative associations. It had the lowest edge number, average degree (8.062), and graph density (0.084), together with the longest average path length (3.783). In contrast, it showed the highest average clustering coefficient (0.600) and modularity (0.582). Positive and negative associations accounted for 86.45% and 13.55% of all edges, respectively. These metrics showed that the *in-situ* network was the sparsest network based on edge number, average degree, and graph density, while also showing the highest modularity and average clustering coefficient among the three networks. The *ex-situ* network contained 99 nodes and 467 edges, including 355 positive and 112 negative associations. Its average degree (9.434), graph density (0.096), and average path length (2.843) were intermediate between the wild and *in-situ* networks, whereas its average clustering coefficient and modularity were both 0.477. Positive associations accounted for 76.02% of all edges, whereas negative associations accounted for 23.98%. Thus, the *ex-situ* network was not the sparsest network; instead, it was characterized by the highest number and proportion of negative co-occurrence associations. These results showed that the wild network had the highest overall connectivity, the *in-situ* network had the sparsest but most modular topology, and the *ex-situ* network had the highest negative-association proportion.

Permutation-based randomization tests (Supplementary Table S9A) showed that the observed networks contained more edges than randomized networks under the same filtering and correlation thresholds. Observed edge numbers were 673, 391, and 467 for the wild, *in-situ*, and *ex-situ* networks, respectively, whereas the corresponding randomized mean edge numbers were 154.3, 178.2, and 203.2. The empirical *P*-value was 0.001 for all three networks, indicating that the observed edge numbers exceeded random expectations. Bootstrap edge-recovery analysis showed that edge-level recovery differed among the three networks. The *ex-situ* network had the highest mean edge-recovery ratio, 0.670, with a 95% confidence interval of 0.497–0.794. The *in-situ* network showed an intermediate recovery ratio of 0.259, with a 95% confidence interval of 0.151–0.453, whereas the wild network showed the lowest recovery ratio of 0.182, with a 95% confidence interval of 0.089–0.333. Thus, although the wild network had the highest number of observed edges, its individual edges showed the lowest bootstrap recovery among the three networks. CLR-transformed network sensitivity analysis (Supplementary Table S9B) was performed to evaluate network patterns after compositional transformation. Compared with the main Spearman networks, CLR-transformed networks had fewer nodes and edges: 81 nodes and 173 edges in the wild group, 83 nodes and 167 edges in the *in-situ* group, and 86 nodes and 197 edges in the *ex-situ* group. In the CLR-transformed networks, negative associations accounted for 22.5% of edges in the wild group, 38.3% in the *in-situ* group, and 38.1% in the *ex-situ* group. These results showed that node and edge numbers were sensitive to compositional-data transformation, whereas the negative-association ratios remained higher in the *in-situ* and *ex-situ* CLR-transformed networks than in the wild CLR-transformed network.

### 3.4 Null-model-inferred community assembly patterns

**Figure 4:**
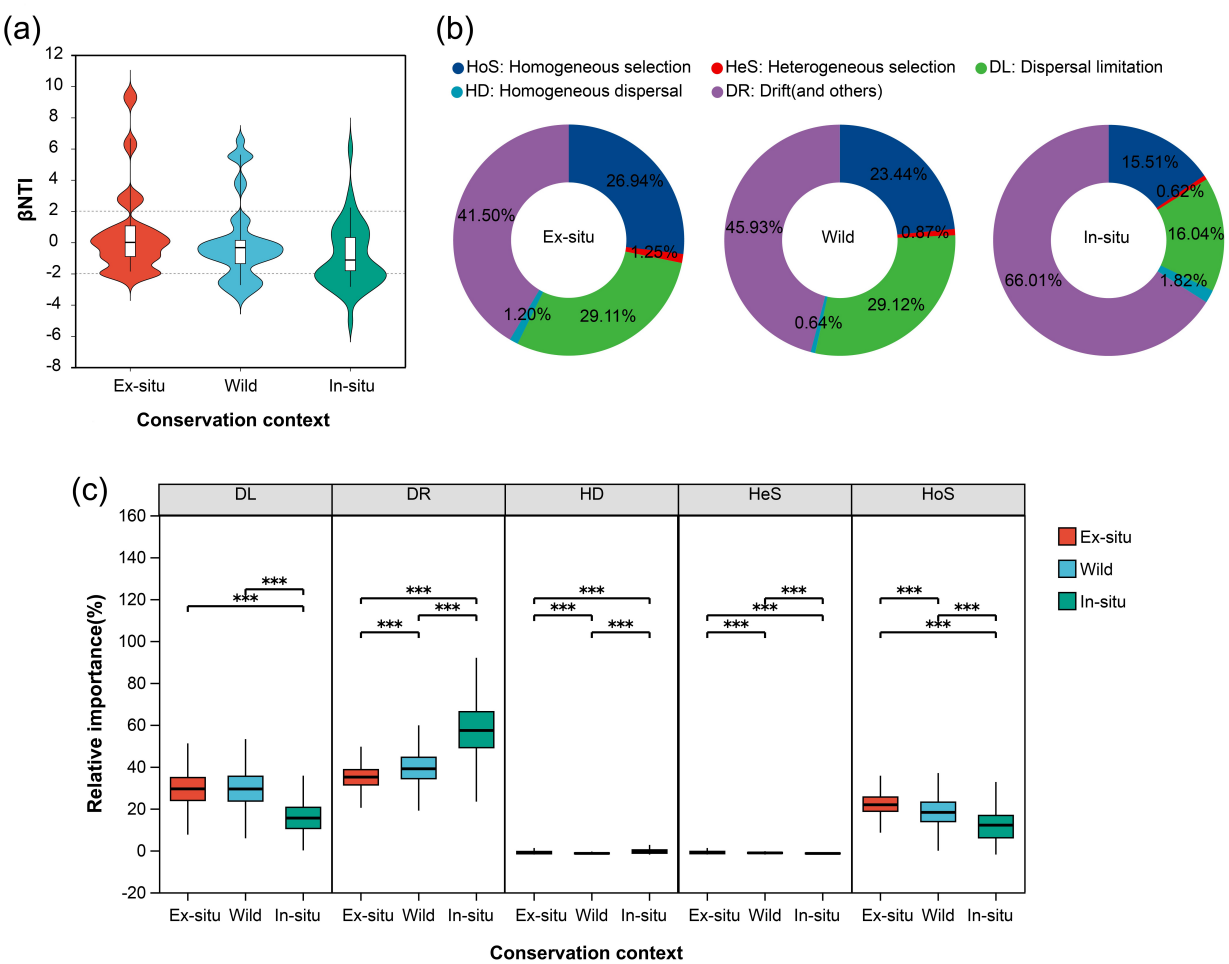
Null-model-inferred assembly categories of gut bacterial communities across conservation contexts. **(a)** βNTI distributions among *ex-situ*, wild, and *in-situ* groups. Dashed horizontal lines indicate βNTI thresholds of +2 and −2. **(b)** Relative contributions of homogeneous selection (HoS), heterogeneous selection (HeS), dispersal limitation (DL), homogenizing dispersal (HD), and drift (DR) in each group. **(c)** Category-level comparisons of relative contributions among conservation contexts. Asterisks indicate significant differences after multiple-comparison adjustment. Assembly categories were inferred using βNTI and RC_bray_ null-model procedures.

Null-model-based community assembly analysis was performed using βNTI and RCbray to assign pairwise comparisons to homogeneous selection, heterogeneous selection, dispersal limitation, homogenizing dispersal, and drift categories. The βNTI distributions showed that most pairwise comparisons were distributed within or near the stochastic range defined by |βNTI| <2, although positive and negative βNTI values outside this range were also observed in each conservation context (Figure 4a).

Across all conservation contexts, stochastic categories accounted for a larger proportion of null-model-inferred assembly than deterministic categories (Figure 4b; Supplementary Table S10A). The stochastic total was highest in the *in-situ* group at 83.87%, followed by the wild group at 75.69% and the *ex-situ* group at 71.81%. Conversely, the deterministic total was highest in the *ex-situ* group at 28.19%, followed by the wild group at 24.31% and the *in-situ* group at 16.13%. Among stochastic categories, drift/undominated processes represented the largest component in all three groups. Drift/undominated processes accounted for 66.01% in the *in-situ* group, 45.93% in the wild group, and 41.50% in the *ex-situ* group. Dispersal limitation accounted for 29.12% in the wild group and 29.11% in the *ex-situ* group, but was lower in the *in-situ* group at 16.04%. Homogenizing dispersal represented a minor component in all groups, accounting for 1.82% in the *in-situ* group, 1.20% in the *ex-situ* group, and 0.64% in the wild group. Among deterministic categories, homogeneous selection contributed more than heterogeneous selection in all conservation contexts. Homogeneous selection accounted for 26.94% in the *ex-situ* group, 23.44% in the wild group, and 15.51% in the *in-situ* group. Heterogeneous selection accounted for only a small proportion of pairwise comparisons, with 1.25% in the *ex-situ* group, 0.87% in the wild group, and 0.62% in the *in-situ* group.

Category-level comparisons further showed significant differences among conservation contexts for most assembly categories (Figure 4c; Supplementary Table S10B). For heterogeneous selection, homogeneous selection, drift/undominated processes, and homogenizing dispersal, all pairwise comparisons among the *ex-situ*, wild, and *in-situ* groups were significant after adjustment. For dispersal limitation, the *ex-situ* and wild groups did not differ significantly, whereas both differed significantly from the *in-situ* group. These results were consistent with the similar dispersal limitation proportions in the *ex-situ* and wild groups and the lower dispersal limitation proportion in the *in-situ* group. Overall, the null-model results showed that stochastic categories accounted for most inferred assembly in all three conservation contexts. The *in-situ* group showed the highest stochastic total and drift/undominated proportion, the *ex-situ* group showed the highest deterministic total and homogeneous selection proportion, and the wild and *ex-situ* groups showed similar dispersal limitation proportions.

## 4 Discussion

In this study, we used the endangered high-altitude fish *Gymnocypris przewalskii*, endemic to the Qinghai-Tibet Plateau, as a model organism to evaluate gut bacterial variation across wild, *in-situ* conservation, and *ex-situ* conservation contexts. Because aquatic organisms are continuously exposed to surrounding water, diet-derived microbes, and environmental microbial reservoirs, their gut bacterial communities can respond sensitively to environmental fluctuation and habitat alteration (Talwar et al., 2018). By integrating bacterial diversity analysis, host morphometric covariate testing, taxonomic profiling, PICRUSt2-based functional prediction, co-occurrence network analysis, and βNTI and RC_bray_-based null-model inference, we evaluated conservation-context-associated bacterial patterns beyond taxonomic composition alone.

### 4.1 Compositional adjustment beneath stable alpha diversity

The gut bacterial communities of *G. przewalskii* showed stable alpha diversity but detectable compositional turnover across conservation contexts. ACE, Chao1, and Shannon indices did not differ significantly among wild, *in-situ*, and *ex-situ* groups, whereas NMDS, PCoA, ANOSIM, and PERMANOVA indicated modest but significant between-group differences (Figures 1a–e; Supplementary Tables S4, S5). This pattern suggests that, across different environmental backgrounds, the gut bacterial communities of *G. przewalskii* maintained overall richness and within-sample diversity while differing in community membership, consistent with compositional adjustment to altered microbial exposure and resource conditions. Similar alpha–beta decoupling has been reported in captive, translocated, and human-managed wildlife, where gut bacterial communities can change in composition without clear reductions in within-sample diversity (McKenzie et al., 2017; Trevelline et al., 2019; West et al., 2019). The non-significant PERMDISP result further supports that this separation was not mainly a dispersion effect, and the association with conservation context persisted after accounting for total length, body weight, and Fulton's condition factor (Supplementary Tables S5, S6).

The taxonomic pattern clarified where this compositional adjustment occurred. Proteobacteria remained dominant in all groups, consistent with its frequent dominance in fish gut communities and broad metabolic flexibility in aquatic environments (Sullam et al., 2012; Wong et al., 2013; Ghanbari et al., 2015). However, dominant genera differed among contexts: wild fish showed higher representation of *Shewanella* and *Vibrio, in-situ* fish showed greater contribution of *Aeromonas* and high *Pseudomonas*, whereas *ex-situ* fish showed higher representation of *Unclassified_f_Enterobacteriaceae, Burkholderia–Caballeronia–Paraburkholderia*, and *Unclassified_f_Rhodobacteraceae* (Figures 2b–e; Supplementary Table S7). Thus, the main bacterial signal was not replacement of the dominant phylum, but redistribution of dominant genera within a broadly conserved phylum-level structure. In aquatic hosts, such genus-level rearrangement is plausible because the gut is continually exposed to water, diet-derived microbes, sediment-associated taxa, and environmental microbial reservoirs (Lokesh and Kiron, 2016; Gao et al., 2023). Differences in water physico-chemistry, diet or feed composition, habitat complexity, and microbial exposure among the three contexts therefore provide ecological background for the observed turnover (Supplementary Tables S2, S3), although these factors cannot be separated from conservation context in the present single-site-per-context design.

### 4.2 Predicted functional profiles indicate shifts in bacterial metabolic potential

The genus-level compositional adjustment observed among wild, *in-situ*, and *ex-situ* fish was accompanied by differences in PICRUSt2-predicted functional profiles (Figures 2f, g; Supplementary Table S8). This finding suggests that bacterial turnover may not represent a purely taxonomic rearrangement, but may also reflect shifts in the metabolic niches occupied by gut bacterial communities under different environmental and feeding backgrounds. In fish, gut bacterial communities are closely linked to digestive ecology because bacteria participate in dietary substrate degradation, enzyme production, carbohydrate and lipid transformation, amino acid metabolism, and energy acquisition (Nayak, 2010; Ray et al., 2012; Ghanbari et al., 2015). Therefore, when conservation contexts differ in natural food resources, formulated feed, water conditions, and microbial exposure, changes in bacterial membership are likely to be accompanied by changes in the predicted metabolic repertoire of the community.

This interpretation is particularly relevant for *G. przewalskii* because the three conservation contexts represent different resource environments rather than only different physical habitats. Wild fish encounter natural riverine food webs and diverse environmental microbial inputs, whereas *in-situ* and *ex-situ* fish experience more standardized feeding and rearing conditions. Such differences can alter the substrates available to gut bacteria and may favor taxa with different capacities for nutrient acquisition and environmental response. Previous fish microbiome studies have shown that diet and rearing environment can restructure gut bacterial communities and modify predicted or measured functional capacities related to digestion, nutrient metabolism, and host energy balance (Egerton et al., 2018; Butt and Volkoff, 2019; Ringø et al., 2022). In this context, correlations between dominant genera and predicted KEGG pathways suggest that shifts involving *Shewanella, Aeromonas, Pseudomonas, Unclassified_f_Enterobacteriaceae, Burkholderia–Caballeronia–Paraburkholderia*, and *Unclassified_f_Rhodobacteraceae* may correspond to differences in microbial resource-use strategies rather than neutral replacement of equivalent taxa.

The predicted functional patterns also provide insight into functional redundancy. Microbial communities can sometimes maintain similar functional capacities despite taxonomic turnover because different taxa share overlapping metabolic traits (Louca et al., 2018). In the present study, however, differential predicted pathways suggest that genus-level turnover was not completely buffered at the functional-potential level. This does not imply that actual metabolic rates changed, but it indicates that bacterial communities associated with different conservation contexts may differ in the range or relative representation of metabolic functions available to the host gut ecosystem. Thus, functional prediction adds an ecological layer to the taxonomic results by suggesting that the gut bacterial response may involve not only changes in bacterial membership, but also shifts in the predicted representation of metabolic capacities under different environmental backgrounds.

These interpretations should be framed as functional hypotheses because PICRUSt2 infers metagenomic potential from 16S rRNA gene profiles rather than directly measuring gene expression or metabolite production. PICRUSt2 improves marker-gene-based prediction by using phylogenetic placement and reference genomes, and NSTI values provide an indicator of prediction reliability (Douglas et al., 2020). The NSTI values in this study support comparative interpretation of the predicted profiles (Supplementary Table S8), but shotgun metagenomics, metatranscriptomics, metabolomics, and enzyme-activity assays will be needed to test whether these predicted metabolic differences translate into realized functional activity in the gut.

### 4.3 Co-occurrence topology reflects context-dependent bacterial organization

The network analysis extends the compositional and predicted functional results by showing that gut bacterial genera were organized differently across conservation contexts (Figure 3; Supplementary Table S9). Relative abundance analysis identifies which taxa differ, whereas co-occurrence topology describes how taxa covary as a community. In microbial ecology, co-occurrence networks are widely used to explore emergent community organization, including connectedness, modularity, and association balance, particularly when community responses are not fully captured by alpha diversity or taxonomic composition alone (Barberán et al., 2012; Berry and Widder, 2014; Matchado et al., 2021). In the present study, the contrasting network structures suggest that the gut bacterial response to different environmental backgrounds involved not only genus-level compositional adjustment, but also reorganization of genus–genus association patterns.

The three networks represented different organizational modes rather than a single directional gradient from wild to *in-situ* to *ex-situ*. The wild network showed dense connectivity and a high proportion of positive associations, a pattern that may be consistent with broader natural microbial inputs and heterogeneous resource conditions. The *in-situ* network was sparser but more modular, suggesting greater compartmentalization of bacterial association groups under semi-managed conditions. In microbial networks, modularity can reflect subsets of taxa that covary more strongly within modules than with the rest of the community, potentially arising from shared habitat preferences, resource partitioning, or common environmental filtering rather than direct interactions (Olesen et al., 2007; Barberán et al., 2012; Berry and Widder, 2014). The *ex-situ* network was distinguished by a higher proportion of negative associations, suggesting stronger separation of bacterial occurrence patterns under a more standardized artificial rearing background. Thus, the main value of the network analysis is not to rank contexts as better or more stable, but to show that each context was associated with a distinct mode of bacterial co-organization.

This network-level perspective refines the interpretation of genus-level turnover. Dominant genera did not merely vary in abundance; they were embedded within different co-occurrence backgrounds. A taxon enriched in one context may occur within a dense association structure, whereas taxa in another context may be distributed across more modular or negatively associated networks. Such differences suggest that conservation-context-associated bacterial reorganization involved both compositional adjustment and changes in the community setting in which dominant genera occurred. Thus, network topology can therefore complement taxonomic profiling by revealing whether dominant genera occur within dense, modular, or negatively associated co-occurrence structures, providing information on community organization that is not captured by relative abundance alone (Matchado et al., 2021; Gao et al., 2022).

Robustness analyses also supported this interpretation while defining its boundaries. Permutation tests showed that observed networks were more structured than randomized networks, bootstrap analysis provided information on edge repeatability, and CLR-transformed sensitivity analysis showed that network size was affected by compositional-data treatment (Supplementary Table S9). Such sensitivity is expected because microbiome network inference depends on data normalization, sparsity, compositionality, and pipeline choices (Kurtz et al., 2015; Liu et al., 2023). Importantly, the broader contrast in association balance, especially the higher negative-association ratio in the *in-situ* and *ex-situ* CLR-transformed networks relative to the wild network, was retained. Therefore, the network results should be interpreted as evidence of context-dependent co-occurrence organization rather than proof of direct bacterial interactions. Correlation-based networks can arise from shared environmental responses, common selection patterns, indirect associations, or compositional constraints, and complex networks may be generated even without direct ecological interactions among taxa (Röttjers et al., 2021; Li et al., 2023). Within these limits, co-occurrence topology provides a useful community-level layer for understanding how gut bacterial organization differs across conservation contexts.

### 4.4 Null-model assembly inference suggests altered balance between source effects and filtering

Null-model assembly analysis provides a process-oriented layer for interpreting the compositional, functional, and network patterns described above. Community assembly theory emphasizes that observed community structure results from the joint influence of niche-based deterministic processes and stochastic processes, rather than from either process alone (Chase and Myers, 2011). In microbial ecology, Stegen et al. (2015) translated this conceptual framework into βNTI- and RC_bray_-based approaches that partition community turnover into categories related to selection, dispersal, and drift-like processes. This framework is particularly relevant for fish gut bacterial communities because aquatic hosts are repeatedly exposed to microbial immigrants from water, diet, sediment, and facility- or habitat-associated reservoirs, while the gut environment filters which taxa persist. Thus, the assembly results in this study can be interpreted as changes in the relative balance between external microbial source effects and local filtering across environmental backgrounds.

Stochastic categories predominated across all groups, suggesting that microbial source input, colonization history, and drift-like dynamics contributed substantially to gut bacterial turnover in *G. przewalskii* (Figure 4; Supplementary Table S10). Zhou and Ning (2017) emphasized that stochasticity is not ecological noise, but an important component of microbial community assembly when dispersal, demographic variation, and weak or fluctuating selection influence community membership. For aquatic fish, such stochasticity is biologically plausible because exposure to external microbial pools is continuous and variable. The stronger drift signal in the *in-situ* group may therefore reflect a semi-managed setting in which environmental microbial inputs remain heterogeneous, while selection imposed by rearing conditions is less uniform than in fully artificial systems. In contrast, the *ex-situ* group showed a stronger homogeneous-selection signal, suggesting a greater role of consistent filtering under standardized feeding, water conditions, and facility management. Stegen et al. (2015) interpreted homogeneous selection as reduced compositional turnover caused by similar environmental pressures across communities, a concept that fits the more uniform *ex-situ* rearing background. Evans et al. (2017) further showed that dispersal and selection jointly influence the reproducibility of microbial assembly outcomes, supporting the broader interpretation that differences in microbial inputs and filtering conditions can shift assembly patterns. In this sense, the *in-situ* and *ex-situ* groups represent different assembly regimes rather than positions along a simple wild-to-captive gradient: the former showed stronger undominated or drift-like turnover, whereas the latter showed a clearer uniform-filtering signal.

Assembly inference further helps connect network topology with the broader ecological processes underlying gut bacterial organization. The *in-situ* group combined a high drift/undominated contribution with a sparse but modular network, suggesting that heterogeneous microbial inputs or weakly coordinated sorting may contribute to compartmentalized co-occurrence organization, whereas the *ex-situ* group combined stronger homogeneous selection with a higher negative-association proportion, consistent with more uniform filtering and stronger separation of bacterial occurrence patterns under standardized artificial rearing conditions (Röttjers et al., 2021; Li et al., 2023). Integrating null-model assembly inference with network topology therefore provides a broader ecological picture than either analysis alone: assembly inference describes how community turnover is partitioned among inferred processes, whereas network topology describes how bacterial genera covary within each context. This combined perspective is useful because microbial co-occurrence networks can reflect common environmental selection, source effects, or shared habitat preferences rather than direct interactions, and null-model comparison can help avoid overinterpreting network structure as biological interaction (Connor et al., 2017). For aquatic hosts, this integration is particularly relevant because gut bacterial communities are continually influenced by water-associated, diet-associated, and habitat-associated microbial reservoirs, which can shape bacterial composition, predicted function, and co-occurrence organization (Spilsbury et al., 2022; Thormar et al., 2024; Zhang et al., 2024). Future work combining replicated environmental microbiome profiling, longitudinal sampling, and experimental manipulation of diet, water chemistry, and microbial exposure will be needed to test whether these inferred assembly regimes reflect specific source effects, filtering processes, or host–environment microbial transmission pathways.

### 4.5 Conservation implications, limitations, and future directions

These findings support the inclusion of gut bacterial ecology as a complementary dimension in conservation assessment for endangered aquatic hosts. Rather than serving as a standalone criterion, microbiome information can help reveal host–environment coupling, dietary transition, microbial source exposure, and management-associated ecological change that may not be captured by conventional demographic, genetic, physiological, or habitat indicators (Hauffe and Barelli, 2019). For *G. przewalskii*, such information may inform feed optimization, water management, habitat enrichment, release preparation, and post-release monitoring, while recognizing that the present observational, single-site-per-context design and 16S-based inference define the results as conservation-context-associated signals rather than causal mechanisms. Future studies should therefore combine replicated and longitudinal sampling with environmental microbiome profiling, metagenomics, metabolomics, and host physiological measurements to test whether these microbial signals can be translated into actionable conservation practice.

## 5 Conclusion

This study demonstrates that gut bacterial communities of the endangered high-altitude fish *G. przewalskii* differed across wild, *in-situ* conservation, and *ex-situ* conservation contexts at multiple ecological levels. Although alpha diversity remained comparable among groups and the broad dominance of Proteobacteria was maintained, beta-diversity patterns, genus-level composition, PICRUSt2-predicted functional profiles, co-occurrence topology, and null-model-inferred assembly categories collectively revealed context-associated bacterial reorganization. These findings suggest that gut bacterial responses to different environmental backgrounds were expressed primarily through compositional adjustment and ecological organization, rather than through a simple loss of bacterial richness or diversity. Among the observed bacterial features, the *in-situ* group shared a high relative abundance of *Pseudomonas* with the wild group but was distinguished by higher network modularity and the strongest stochastic or drift contribution. In contrast, the *ex-situ* group showed a higher proportion of negative associations and a stronger homogeneous-selection signal. Within the constraints of an observational, single-site-per-context design, gut bacterial profiling may therefore complement traditional conservation indicators by providing information on host–environment microbial coupling, dietary and environmental microbial exposure, and management-associated bacterial organization. Future replicated, longitudinal studies integrating environmental microbiomes and multi-omics approaches are needed to test whether these context-associated bacterial signals translate into functional consequences for host health, adaptation, and conservation planning.

## Data Availability

The datasets presented in this study can be found in online repositories. The names of the repository/repositories and accession number(s) can be found in the article/Supplementary material.
