# Supplementary material for: From assembly inference to co-occurrence organization: conservation contexts are associated with gut bacterial variation in the endangered Gymnocypris przewalskii
Source: Front Microbiol. 2026 Jul 9;17:1875592. doi: 10.3389/fmicb.2026.1875592 (PMC13391935; doi:10.3389/fmicb.2026.1875592)
Supplement: Supplementary file 1 [file Data_Sheet_1.pdf]

Supplementary Tables S1–S10

Supplementary Table Index

Table	Title
Supplementary Table S1	Sampling design, host morphometric characteristics, and age/developmental-stage data
Supplementary Table S2	Water physicochemical parameters
Supplementary Table S3	Diet/feed composition
Supplementary Table S4	Alpha diversity indices and Kruskal-Wallis tests
Supplementary Table S5	Beta diversity statistics: ANOSIM, PERMANOVA, PERMDISP
Supplementary Table S6	Host morphometric statistical tests and covariate PERMANOVA
Supplementary Table S7	Taxonomic composition / dominant phyla and genera
Supplementary Table S8	PICRUSt2 predicted pathways and NSTI values
Supplementary Table S9	Network topology and robustness
Supplementary Table S10	Null-model assembly categories

Supplementary Table S1. Sampling design, host morphometric characteristics, and age/developmental-stage data.

Group	Number of fish	Sampling context	Sex	Age/developmental information	Total length (cm), mean ± SD (range)	Body weight (g), mean ± SD (range)	Condition factor, mean ± SD (range)	Sampling rationale
Wild	9	Buha River inflow of Qinghai Lake	Male	Sexually mature adult; approximate adult age range estimated from body size, sexual maturity, and field observations; exact individual age unavailable	24.17 ± 2.11 (20.0–27.5)	81.44 ± 18.91 (48–116)	0.569 ± 0.027 (0.525–0.601)	Only sexually mature males were destructively sampled to reduce sex/reproductive-status variation and avoid impacts on reproductive females of this endangered species.
In-situ	9	Qinghai Lake Naked Carp Rescue and Conservation Center	Male	Sexually mature adult; 3 years based on rescue-center breeding/management records	31.39 ± 2.43 (27.0–35.5)	251.11 ± 49.36 (180–350)	0.808 ± 0.067 (0.668–0.914)	Same criterion applied across groups.
Ex-situ	9	Ex-situ breeding facility in Hebei Province	Male	Sexually mature adult; 3 years based on breeding-farm management records	29.82 ± 2.98 (26.5–35.2)	233.67 ± 52.03 (168–311)	0.879 ± 0.107 (0.688–0.986)	Same criterion applied across groups.

Supplementary Table S2. Water physicochemical parameters across conservation contexts.

Group	n	Water temp (°C), mean ± SD	Water temp range	pH, mean ± SD	pH range	Dissolved oxygen (mg/L), mean ± SD	Dissolved oxygen range	Total nitrogen (mg/L), mean ± SD	Total nitrogen range	Total phosphorus (mg/L), mean ± SD	Total phosphorus range
Wild	9	13.88 ± 0.31	13.30–14.20	8.14 ± 0.11	8.01–8.27	9.30 ± 0.22	9.00–9.60	2.172 ± 0.233	1.910–2.480	0.172 ± 0.017	0.150–0.198
In-situ	9	17.97 ± 0.56	17.40–19.00	8.70 ± 0.08	8.62–8.80	4.48 ± 0.26	4.03–4.88	2.143 ± 0.095	2.030–2.280	0.263 ± 0.007	0.251–0.275
Ex-situ	9	16.39 ± 1.00	15.10–17.80	8.07 ± 0.13	7.80–8.20	7.37 ± 1.14	5.98–8.90	4.006 ± 1.001	2.856–5.958	0.304 ± 0.293	0.044–0.824

Supplementary Table S3. Diet/feed composition across conservation contexts.

Group	Food/feed type	Main food or feed ingredients	Key nutritional composition	Feeding regime	Notes/information source
Wild	Natural food resources	Aquatic insects, cladocerans, copepods, algae, and organic detritus	Approximate nutritional ranges of mixed natural food items: crude protein 5–65%; crude fat 1–12%; ash 1.42–50%; carbohydrates 8–30%	Natural foraging; no artificial feeding	Exact individual dietary intake could not be quantified for wild fish; composition is reported as an approximate natural-food range.
In-situ	Specialized formulated feed for naked carp	Wheat (wheat middlings), soybean meal, peanut meal, rapeseed meal, fish meal, soybean lecithin oil, minerals, vitamins, oligosaccharides, and amino acids	Crude protein ≥40.0%; crude fat ≥4.0%; crude fiber ≤8.0%; crude ash ≤15.0%; total phosphorus ≥1.2%; lysine ≥2.1%; moisture ≤12.5%	Automatic feeding machine; 3 times daily	Information obtained from conservation management and feeding records.
Ex-situ	Commercial formulated feed	Fish meal, soybean meal, wheat flour, fish oil, soybean oil, yeast polysaccharides, enzyme preparations, vitamins, mineral supplements, probiotics, taurine, lysine, and methionine	Crude protein ≥38.0%; crude fat ≥9.0%; calcium ≥1.0%; total phosphorus ≥0.8%; lysine ≥2.0%; sodium chloride 0.3–1.5%; crude ash ≤15.0%; crude fiber ≤8.5%; moisture ≤14.0%	Manual scheduled feeding; 3 times daily	Information obtained from feeding records and available feed information.

Supplementary Table S4. Alpha diversity indices and Kruskal-Wallis tests.

Alpha diversity index	Statistical test	P value	Result
ACE	Kruskal-Wallis	0.4281	Not significant
Chao1	Kruskal-Wallis	0.3327	Not significant
Shannon	Kruskal-Wallis	0.4835	Not significant

Supplementary Table S5. Beta diversity statistics: ANOSIM, PERMANOVA, and PERMDISP.

Analysis	Statistic	Value	P value
Analysis of similarities (ANOSIM)	R	0.1762	0.01
Permutational multivariate analysis of variance (PERMANOVA)	R squared	0.1428	0.012
Homogeneity of multivariate dispersion (PERMDISP)	F	0.127	0.898

Note. These statistics represent the primary beta-diversity analysis reported in the main text and Figure 1. ANOSIM, PERMANOVA, and PERMDISP were used to evaluate overall group separation and homogeneity of multivariate dispersion among wild, in-situ, and ex-situ conservation contexts.

Supplementary Table S6A. Assumption checks and statistical tests for host morphometric traits.

Trait	Shapiro-Wilk normality by group	Levene F	Levene P	ANOVA F	ANOVA P	Kruskal-Wallis H	Kruskal-Wallis P	Eta squared
Total length	Ex-situ: W = 0.934, P = 0.5222; In-situ: W = 0.984, P = 0.9812; Wild: W = 0.957, P = 0.7632	0.5908	0.5617	20.27	6.99e-06	16.5	0.0002614	0.6281
Body weight	Ex-situ: W = 0.941, P = 0.5896; In-situ: W = 0.961, P = 0.8118; Wild: W = 0.928, P = 0.4644	3.246	0.0565	42.76	1.23e-08	17.66	0.0001463	0.7809
Condition factor	Ex-situ: W = 0.874, P = 0.1368; In-situ: W = 0.938, P = 0.5651; Wild: W = 0.920, P = 0.3947	2.729	0.0856	42.3	1.36e-08	18.56	9.31e-05	0.779

Supplementary Table S6B. Tukey post-hoc comparisons for host morphometric traits.

Trait	Comparison	Mean difference	Adjusted P value	Lower 95% CI	Upper 95% CI	Significant
Total length	Ex-situ - In-situ	1.567	0.402	-1.413	4.547	No
Total length	Ex-situ - Wild	-5.656	0.0002	-8.636	-2.675	Yes
Total length	In-situ - Wild	-7.222	0	-10.2	-4.242	Yes
Body weight	Ex-situ - In-situ	17.44	0.6675	-32.96	67.85	No
Body weight	Ex-situ - Wild	-152.2	0	-202.6	-101.8	Yes
Body weight	In-situ - Wild	-169.7	0	-220.1	-119.3	Yes
Condition factor	Ex-situ - In-situ	-0.0714	0.128	-0.1596	0.0167	No
Condition factor	Ex-situ - Wild	-0.31	0	-0.3981	-0.2218	Yes
Condition factor	In-situ - Wild	-0.2385	0	-0.3267	-0.1504	Yes

Supplementary Table S6C. Bray-Curtis PERMANOVA models testing host morphometric covariates and conservation context.

Model	Term	df	SS	F	R squared	Partial R squared	P value
Bray-Curtis ~ Group	Group only	2	1.37	2.225	0.1564	0.1564	0.0057
Bray-Curtis ~ Length + Weight + K	Length + Weight + K	3	1.233	1.256	0.1408	0.1408	0.1624
Bray-Curtis ~ Length + Weight + K + Group	Group after Length + Weight + K	2	1.264	2.12	0.1443	0.168	0.0063
Sequential covariate test	Length	1	0.6423	1.979	0.0733	0.0733	0.037
Sequential covariate test	Weight after Length	1	0.3114	0.9576	0.0356	0.0384	0.4613
Sequential covariate test	K after Length + Weight	1	0.2794	0.854	0.0319	0.0358	0.5646

Note. Supplementary Table S6C reports Bray–Curtis PERMANOVA models used for host-morphometric covariate assessment. The group-only model in this table was included as a reference model within the covariate-analysis workflow and should be interpreted together with the covariate-adjusted models.

Supplementary Table S7. Taxonomic composition / dominant phyla and genera.

Taxonomic level	Taxon	Wild (%)	In-situ (%)	Ex-situ (%)
Phylum	Proteobacteria	76.81	80.97	78.49
Phylum	Firmicutes	11.41	7.75	3.27
Phylum	Bacteroidetes	2.67	4.84	2.85
Phylum	Actinobacteria	2.68	2.79	4.65
Phylum	Chloroflexi	0.93	0.68	5.01
Phylum	Acidobacteria	1.68	0.75	1.18
Phylum	Fusobacteria	1.90	0.67	0.15
Phylum	Cyanobacteria	0.13	0.07	1.41
Phylum	Spirochaetes	0.07	0.12	1.12
Phylum	Patescibacteria	0.32	0.42	0.29
Phylum	Others	1.39	0.95	1.59
Genus	*Pseudomonas*	27.00	28.35	18.95
Genus	*Unclassified_f_Enterobacteriaceae*	6.16	21.44	15.52
Genus	*Shewanella*	17.01	2.57	0.30
Genus	*Burkholderia-Caballeronia-Paraburkholderia*	2.27	2.94	11.07
Genus	*Aeromonas*	4.17	10.46	0.12
Genus	*Ralstonia*	2.22	2.71	4.85
Genus	*Staphylococcus*	6.99	0.75	0.66
Genus	*Vibrio*	7.74	0.05	0.10
Genus	*Unclassified_f_Rhodobacteraceae*	0.37	0.19	7.25
Genus	*Acinetobacter*	1.07	1.64	1.80
Genus	Others	25.01	28.90	39.39

Supplementary Table S8A. PICRUSt2-predicted pathways summarized for functional profiling.

PICRUSt2	Value or category
Number of differentially enriched predicted KEGG pathways/modules	14
Predicted KEGG modules included in genus-pathway correlation analysis	M00373, M00020, M00615, M00169, M00131, M00545, M00595, M00097, M00563, M00596, M00030, M00066, M00091, M00835
Broad functional categories represented	Carbohydrate metabolism; energy metabolism; lipid metabolism; amino acid metabolism; terpenoid and polyketide metabolism; secondary metabolite biosynthesis; xenobiotic biodegradation and metabolism

Supplementary Table S8B. Weighted NSTI summary for PICRUSt2 prediction reliability.

Group	n	Mean	SD	Minimum	Maximum
Wild	9	0.0787	0.029	0.0451	0.121
In-situ	9	0.071	0.069	0.0203	0.2069
Ex-situ	9	0.0848	0.0516	0.029	0.1677
Overall	27	0.0782	0.0507	0.0203	0.2069

Supplementary Table S8C. Individual sample-level weighted NSTI values.

Sample ID	Group	Weighted NSTI
wild1	Wild	0.1179
wild2	Wild	0.06438
wild3	Wild	0.08347
wild4	Wild	0.06211
wild5	Wild	0.121
wild6	Wild	0.05569
wild7	Wild	0.0547
wild8	Wild	0.1043
wild9	Wild	0.04507
ex-situ1	Ex-situ	0.1115
ex-situ2	Ex-situ	0.1388
ex-situ3	Ex-situ	0.1677
ex-situ4	Ex-situ	0.04908
ex-situ5	Ex-situ	0.1277
ex-situ6	Ex-situ	0.03564
ex-situ7	Ex-situ	0.02903
ex-situ8	Ex-situ	0.05137
ex-situ9	Ex-situ	0.05237
in-situ1	In-situ	0.1743
in-situ2	In-situ	0.03145
in-situ3	In-situ	0.04208
in-situ4	In-situ	0.04746
in-situ5	In-situ	0.03202
in-situ6	In-situ	0.02034
in-situ7	In-situ	0.2069
in-situ8	In-situ	0.032
in-situ9	In-situ	0.05268

Supplementary Table S9A. Co-occurrence network topology and robustness metrics.

Group	Nodes	Edges	Positive edges	Negative edges	Positive ratio (%)	Negative ratio (%)	Density	Average degree	Average path length	Average clustering coefficient	Modularity	Random mean edges	Permutation empirical P	Bootstrap edge recovery ratio (95% CI)
Wild	95	673	643	30	95.54	4.46	0.151	14.168	2.506	0.574	0.383	154.3	0.001	0.182 (0.089–0.333)
In-situ	97	391	338	53	86.45	13.55	0.084	8.062	3.783	0.600	0.582	178.2	0.001	0.259 (0.151–0.453)
Ex-situ	99	467	355	112	76.02	23.98	0.096	9.434	2.843	0.477	0.477	203.2	0.001	0.670 (0.497–0.794)

Supplementary Table S9B. CLR-transformed network sensitivity analysis.

Group	Retained genera	Network nodes	Edges	Positive edges	Negative edges	Positive ratio (%)	Negative ratio (%)	Density	Average degree	Threshold
Wild	100	81	173	134	39	77.5	22.5	0.053	4.272	$\|\rho\| > 0.6$; $P < 0.01$
In-situ	100	83	167	103	64	61.7	38.3	0.049	4.024	$\|\rho\| > 0.6$; $P < 0.01$
Ex-situ	100	86	197	122	75	61.9	38.1	0.054	4.581	$\|\rho\| > 0.6$; $P < 0.01$

Supplementary Table S10A. Null-model assembly categories shown in Figure 4b.

Group	Homogeneous selection (%)	Heterogeneous selection (%)	Deterministic total (%)	Dispersal limitation (%)	Homogenizing dispersal (%)	Drift (%)	Stochastic total (%)
Ex-situ	26.94	1.25	28.19	29.11	1.2	41.5	71.81
Wild	23.44	0.87	24.31	29.12	0.64	45.93	75.69
In-situ	15.51	0.62	16.13	16.04	1.82	66.01	83.87

Supplementary Table S10B. Pairwise comparisons among conservation contexts for each null-model assembly category.

Assembly category	Pairwise comparison	Adjusted P value	Cohen's d	Significance
Dispersal limitation (DL)	Ex-situ vs In-situ	4.56e-188	1.629	***
Dispersal limitation (DL)	Ex-situ vs Wild	0.6095	-0.053	NS
Dispersal limitation (DL)	Wild vs In-situ	6.29e-190	1.628	***
Drift/undominated (DR)	Ex-situ vs In-situ	8.86e-275	-2.428	***
Drift/undominated (DR)	Wild vs Ex-situ	2.22e-39	0.621	***
Drift/undominated (DR)	Wild vs In-situ	3.79e-209	-1.820	***
Homogenizing dispersal (HD)	In-situ vs Ex-situ	2.26e-33	0.551	***
Homogenizing dispersal (HD)	Wild vs Ex-situ	2.47e-37	-0.757	***
Homogenizing dispersal (HD)	Wild vs In-situ	5.74e-109	-1.262	***
Heterogeneous selection (HeS)	Ex-situ vs In-situ	8.25e-60	0.913	***
Heterogeneous selection (HeS)	Ex-situ vs Wild	3.22e-11	0.518	***
Heterogeneous selection (HeS)	Wild vs In-situ	1.65e-73	0.875	***
Homogeneous selection (HoS)	Ex-situ vs Wild	8.44e-40	0.599	***
Homogeneous selection (HoS)	In-situ vs Ex-situ	2.93e-183	-1.614	***
